# Colour discrimination deficit in REM sleep behavior disorder: an analysis of dopaminergic depletion, cognition, and brain morphology

**DOI:** 10.1007/s00702-025-02936-w

**Published:** 2025-06-06

**Authors:** Filip Havlik, Christiane Mala, Petr Dusek, Josef Mana, Veronika Ibarburu Lorenzo y Losada, Simona Dostálová, Jiří Nepožitek, Pavla Peřinová, Evžen Růžička, Radim Krupička, Karel Šonka, Ondrej Bezdicek

**Affiliations:** 1 Department of Neurology and Centre of Clinical Neuroscience, First Faculty of Medicine, Charles University and General University Hospital in Prague, Prague, Czech Republic; 2 Department of Biomedical Informatics, Faculty of Biomedical Engineering, Czech Technical University in Prague, Prague, Czech Republic

**Keywords:** Farnsworth-Munsell 100-Hue Test, Vision, Cognitive functions, Synucleinopathy, REM sleep behavior disorder

## Abstract

Isolated REM sleep behavior disorder (iRBD) is associated with impaired colour discrimination, cognitive deficits and morphological changes. This study evaluates whether colour discrimination deficits in iRBD are mediated by cognitive functions or related to dopaminergic denervation and brain morphology. A sample of 73 patients with iRBD and 77 controls underwent neuropsychological assessment, and colour discrimination assessment using the Farnsworth Munsell 100 Hue Test, DAT-SPECT, and MRI. The data were analyzed using multiple regression, mediation analysis, and voxel-based morphometry. Significant between-group differences were found in total colour discrimination as well as in the red-yellow spectrum. The association between iRBD and performance in the yellow-green spectrum was mediated by cognitive functions, as measured by the Montreal Cognitive Assessment. In controls, a positive correlation between the yellow-green spectrum and the left inferior frontal gyrus was observed compared to patients, however, this association was largely driven by a single data point. The performance in the green–blue spectrum was associated with the activity of dopamine transporters in the caudate nucleus. No interactions were found for total colour discrimination in any analysis. The present findings demonstrate a colour vision deficit in iRBD, which is not directly linked to any of the proposed potential explanatory mechanisms.

## Introduction

Isolated rapid eye movement (REM) sleep behavior disorder (iRBD) is a prodromal stage of alpha-synucleinopathies such as Parkinson’s disease (PD) or dementia with Lewy bodies (DLB). iRBD is associated with neuropsychiatric, cognitive and olfactory deficits (Youn et al. 2016), but extensive literature provides evidence of significant impairment in visual processing as well (Marques et al. 2010; Postuma et al. 2015; Li et al. 2019).

Changes in visual processing in iRBD deviate from normal ageing approximately five years before the clinical diagnosis of neurodegeneration (Dall’Antonia et al. 2018; Fereshtehnejad et al. 2019). These changes are associated with cognitive impairment and can predict further cognitive decline (Rahayel et al. 2018; Li et al. 2019; Zarkali et al. 2021). Therefore, vision abnormalities represent a promising marker for cognitive deficits in synucleinopathies and a potential target for symptomatic treatment. However, the pathophysiological basis, particularly in colour discrimination, and its relationship to other factors such as cognitive functions, dopaminergic and morphological changes remain under debate.

Poorer performance in colour discrimination in iRBD and PD (Postuma et al. 2009; Dusek et al. 2019; Fereshtehnejad et al. 2019; Li et al. 2019; Kim et al. 2024) has been explained by retinal disruption, such as ganglion cell layer loss, macular thinning or neurodegeneration of amacrine cells (Polo et al. 2016; Ortuno-Lizaran et al. 2020), alterations in visual pathways, including white matter integrity in the corona radiata and visual stream (Unger et al. 2010) or widespread cortical and subcortical brain changes (Rahayel et al. 2018). Additionally, the role of higher-level cognition has also been considered (Bertrand et al. 2012).

Given that dopamine neurons are severely affected in PD, the involvement of dopamine has been proposed in iRBD as well (Kordower et al. 2013; Colzato et al. 2014). While both dopaminergic cells in the retina and brain may be impacted, some researchers argue that brain dopaminergic denervation is particularly crucial. For instance, Colzato et al. (2014) hypothesizes that deficits in executive functions and colour discrimination share a common cause, i.e. dopaminergic denervation, suggesting that colour discrimination could serve as a potential proxy for the “central dopaminergic state”. However, the authors do not comment on how colour vision should be related to dopamine and executive functions, whether striatal dopamine depletion is directly responsible for both deficits, or whether striatal dopamine depletion causing only cognitive deficits and retinal dopamine depletion causing colour deficits are separate but correlated. Furthermore, others dispute this idea (Marques et al. 2010; Bertrand et al. 2012), and the lack of a significant correlation between colour discrimination and nigrostriatal dopaminergic degeneration in PD also weakens this hypothesis (Muller et al. 1998). To our knowledge, only one study has examined this association in iRBD and, similarly, found no significant correlation (Dusek et al. 2019). Overall, reports on this topic are sparse, and the available studies present inconclusive results.

Findings on the relationship between colour discrimination and cognitive functions are equally inconsistent. In both non-clinical populations and those with alpha-synucleinopathies, colour discrimination has been associated with executive functions (Bertrand et al. 2012; Colzato et al. 2014), memory (Bertrand et al. 2012), visuoconstructive and visuospatial functions (Bertrand et al. 2012; Matar et al. 2019), cognitive screening tests (Li et al. 2019), and motor symptoms (Muller et al. 1998).

Given these conflicting or equivocal results, this study aims to assess colour discrimination in iRBD combined with a comprehensive neuropsychological battery, dopamine transporter single-photon emission computed tomography (DAT-SPECT) and voxel-based morphometry (VBM). Specifically, the study seeks to answer three key questions: (1) whether cognitive functions account for differences in colour discrimination between iRBD and controls, (2) whether the colour discrimination deficit in iRBD is related to dopaminergic denervation, and (3) whether performance in colour discrimination can be explained by morphometric parameters of cerebral grey and white matter.

## Methods

### Causal assumptions and interpretation criteria

**Fig. 1:**
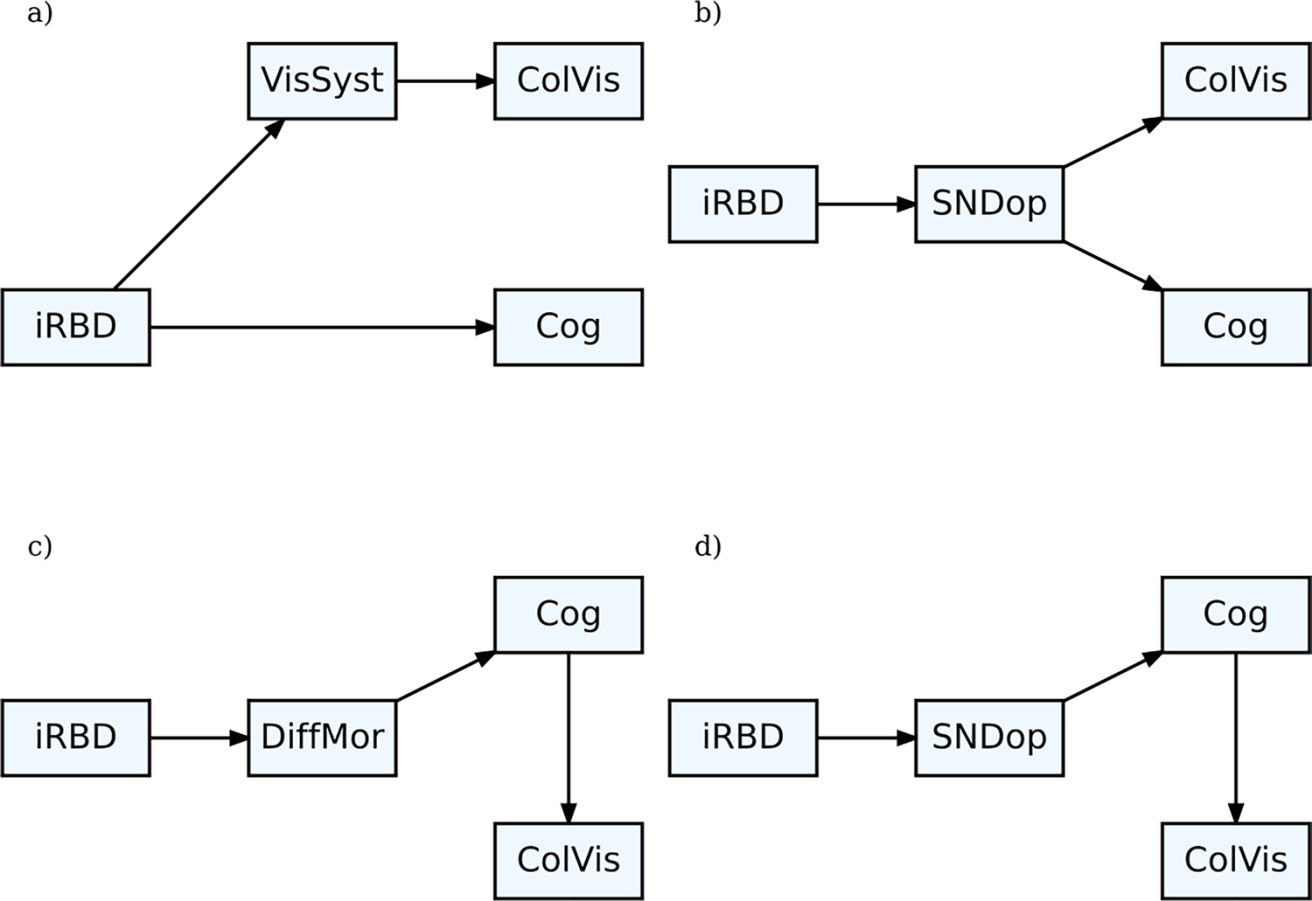
Competing causal assumptions *Note*. In all DAGs represented in this figure we assume that demographic variables age, sex and education level constitute common causes of iRBD, cognition, colour discrimination and sample selection and were thus appropriately adjusted for in all analyses. *VisSyst* = visual system; *ColVis* = colour vision; *Cog* = cognition; *SNDop* = substantia nigra dopamine; *DiffMorf* = diffuse morphological changes

For illustrative purposes, we propose several competing causal assumptions based on previous studies. These assumptions include: (a) the colour discrimination deficit is caused by a structural disturbance in the visual system (Unger et al. 2010; Polo et al. 2016), (b) the colour discrimination deficit is driven by a cognitive deficit resulting from diffuse morphological changes (Bertrand et al. 2012), (c) both the colour discrimination and cognitive deficits are due to reduced dopamine levels in the basal ganglia (Colzato et al. 2014), (d) the colour discrimination deficit is driven by a cognitive deficit resulting from reduced dopamine levels in the basal ganglia (Colzato et al. 2014). See DAGs (directed acyclic graphs) in Fig. 1 for further details.

Given the nature of the data and research questions, several a priori criteria were established to interpret the statistical analysis results. For the first research question, the criterion for a meaningful mediation effect of cognitive functions is the presence of statistically significant results in at least two psychological tests within the same cognitive or motor domain, or a significant result in a test that measures multiple domains (e.g., MoCA). This criterion reflects the number of tests and the overlap of cognitive domains they assess. For the second and third research questions, any significant association will be deemed substantively meaningful, as the mechanisms affecting performance on the Farnsworth-Munsell 100-Hue Test (FM-100) may differ. Colour discrimination will serve as a proxy for the central dopaminergic state if at least one FM-100 score is significantly associated with DAT-SPECT values of the caudate nucleus and putamen, or striatum.

### Study sample

**Fig. 2:**
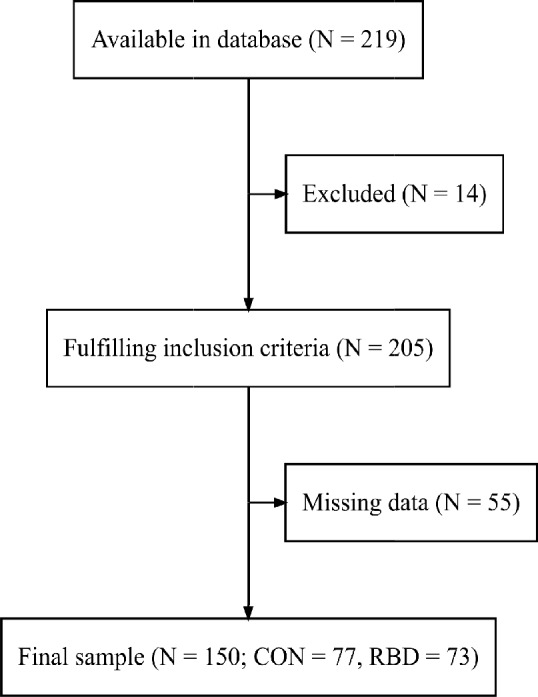
Inclusion flowchart

Both groups were sampled using a convenience sampling method between 2015 and 2024 as part of a larger study at the General University Hospital in Prague. The patient group was recruited from the Movement Disorders Center and Sleeping Laboratory at the Department of Neurology. The control group (CON) was recruited via social media and web advertisement from the general community. Both groups underwent complex neurological and neuropsychological examination including polysomnographic examination. Only patients with iRBD diagnosed according to the International Classification of Sleep Disorders, third edition (American Academy of Sleep Medicine 2014) without clinical signs of overt parkinsonism or dementia were included. Furthermore, the presence of head injury, narcolepsy, severe untreated psychiatric symptoms (e.g. depression or mania), drug-induced RBD, or focal brain lesion indicative of secondary RBD were exclusion criteria. The control group, enrolled to be comparable in terms of age, sex and education, met all the above criteria, and, additionally, the presence of RBD was excluded in all controls by polysomnography and had to be free of mild cognitive impairment (MCI) according to level 1 (Litvan et al. 2012). See Fig. 2 for an inclusion flow chart.

### Neuropsychological assessment

All participants were screened for cognitive deficit, neuropsychiatric and motor symptoms using the Montreal Cognitive Assessment (MoCA)(Nasreddine et al. 2005; Kopecek et al. 2016), Beck Depression Inventory, Second Edition (BDI-II)(Beck et al. 1996; Ciharova et al. 2020), State-Trait Anxiety Inventory (STAI)(Spielberger et al. 1983), and Movement Disorders Society-Unified Parkinson’s Disease Rating Scale, part III (MDS-UPDRS-III)(Goetz et al. 2008). To assess specific cognitive domains, participants underwent a neuropsychological battery comprising the following tests: Rey Auditory Verbal Learning Test (RAVLT; memory)(Bezdicek et al. 2014b), Memory Binding Test (MBT; memory)(Buschke 2014), abbreviated Memory for Intentions Screening Test (MIST; memory)(Raskin et al. 2010; Bezdicek et al. 2014a), Trail Making Test (TMT; attention/working memory, executive functions)(Bezdicek et al. 2012), Letter-Number Sequencing from Wechsler Adult Intelligence Scale, Third Revision (LNS; attention/working memory)(Wechsler 2010), Prague Stroop Test (PST; executive functions)(Bezdicek et al. 2015), Verbal fluency (VF; language/executive functions)(Nikolai et al. 2015), Clock Drawing Test (CDT; visuospatial functions)(Kopecek et al. 2016), MoCA Cube (visuospatial functions)(Kopecek et al. 2016), Grooved Pegboard Test (GPT; the speed of processing)(Kløve 1963), and Symbol Digit Modalities Test (SDMT; the speed of processing)(Smith 1982).

### Colour discrimination assessment

Binocular colour discrimination was assessed using the Farnsworth-Munsell 100-Hue Test (Farnsworth 1957), which consists of 85 colour caps. These caps represent different hue grades while maintaining constant saturation and lightness. The caps are organized into four trays, with the first tray containing 22 caps and the subsequent trays containing 21 caps each. Each tray includes two fixed reference caps at opposite ends, serving as anchors for the colour-sorting task. During the test, the examiner presents the trays to the examinee, who is instructed to arrange the caps in a specific order. This order should form a smooth transition of hues between the two reference caps, replicating the natural progression of the colour spectrum. Once the test is completed, the examiner assesses the results by calculating deviations from the correct colour sequence. Each misplaced cap incurs a penalty, and the total error score (TES) indicates the overall degree of chromatic discrimination impairment. In addition, the test generates four subscores, one for each spectrum: red-yellow (RY), yellow-green (YG), green–blue (GB), and blue-red (BR). The order of these subscores also corresponds to the order of administration. The FM-100 test was administered in a dark room without windows under artificial lighting provided by a 1440 lm, 6500 K incandescent lamp.

### MRI data acquisition

The examination was performed on a 3 T MRI scanner (Siemens Skyra 3 T, Siemens Healthcare, Erlangen, Germany) with a 32-channel head coil. Morphometry analysis was performed on T1-weighted 3D Magnetization-Prepared Rapid Acquisition with Gradient Echo (MPRAGE) images in the axial plane with the following acquisition parameters: repetition time (TR), 2200 ms; echo time (TE), 2.4 ms; inversion time (TI) 900 ms; flip angle (FA) 8°; field of view (FOV) 230 × 197 × 176 mm; spatial resolution 1 × 1x1 mm^3^.

### Dopamine transporter imaging

All patients with iRBD underwent dopamine transporter single photon emission computed tomography (DAT-SPECT) to assess the integrity of nigrostriatal dopaminergic functioning. This examination utilized [123I]−2-b-cabomethoxy-3b-(4-iodophenyl)-N-(3-fluoropropyl) nortropane (DaTscan®, GE Healthcare) as the radiopharmaceutical, following the procedure guidelines of the European Association of Nuclear Medicine (Darcourt et al. 2010) and employing the common acquisition and reconstruction parameters described in detail previously (Dusek et al. 2019). Semi-quantitative analysis of images was performed using the DaTQUANT V2 software. The specific to non-displaceable binding ratios (SBRs) were determined in the bilateral striatum, caudate, and putamen using the formula (nucleus uptake – background uptake)/background uptake, with bilateral occipital lobes serving as the background reference region. Following the methodology of Arnaldi et al. (2024), DAT-SPECT scans were classified as normal (z-score > −1) or abnormal (z-score ≤ −1) based on the putaminal SBR from the hemisphere with the lower tracer binding. Mean striatal, caudate, and putamen SBRs from both hemispheres were analyzed in the current study.

### Voxel based morphometry

The preprocessing and segmentation of T1-weighted images were conducted using the Computational Anatomy Toolbox (CAT12; version 12.8.2), implemented in Statistical Parametric Mapping software (SPM, version 7771) within Matlab. A visual quality check was performed by reviewing one slice of each brain to identify obvious artefacts in the scans and inaccurately oriented images. The homogeneity of the data was assessed by applying the CAT batch of data quality. The segmentation quality of each image was accepted at a minimum rating of C + across all quality parameters. The modulated, normalized grey matter segments were smoothed using a Gaussian kernel with an 8 mm3 full width at half maximum to perform voxel-based morphometry (VBM).

### Statistical analysis

Colour discrimination was assessed using the FM-100, where a higher score indicates a greater degree of deficit. Cognitive indices, in which higher scores reflect better performance (e.g., recall scores in memory tests), were multiplied by negative ones before being entered into the analysis. As a result, positive regression coefficients suggest a positive association between the quality of visual discrimination and cognitive performance on any given test.

#### Group differences in colour discrimination

The first step of the analysis involved establishing foundational knowledge about colour discrimination and its relationship to group characteristics. To achieve this, generalized linear models (GLM) and Tobit models were employed, with group, age, education, and sex as predictors and FM-100 scores as the outcomes. The GLM with gamma-distributed errors and a log link function was used to address the positively skewed and non-negative nature of the FM-100 (TES) score. All FM-100 subscores representing a deficit for each colour spectrum were censored at zero. To account for this censoring, the Tobit model with a Gaussian distribution and robust standard errors implemented in the R package Survival (version 3.6.4) (Therneau 2024) was used.

#### Effect of iRBD on colour discrimination through cognition

As part of the mediation analysis, two sets of regression analyses were conducted. The first set aimed to examine the relationship between the mediator and the outcome, while the second set assessed the relationship between the group and the outcome, accounting for the mediator. In the first set, psychological variables were treated as outcomes, with group, age, sex, and education as predictors. GLM with an inverse Gaussian distribution was used for positively skewed data. For MDS-UPDRS and MoCA, the Tobit model was employed, while ordinary least squares (OLS) regression was used in the other cases. The second set of analyses used the same models as those employed in the analysis of group differences in colour discrimination. However, an interaction term group × psychological variables (with the group represented by an indicator variable, i.e., CON = 0, iRBD = 1) was added to the models, given that the proposed underlying mechanism for the colour discrimination deficit in iRBD is based on disease pathology. Both sets of regressions were then entered into the mediation analysis performed in the Mediation package (version 4.5.0) (Tingley et al. 2014).

#### Association of colour discrimination with dopamine transporter imaging

A series of GLM and Tobit regressions was performed, this time exclusively on the iRBD group. The choice of model followed the same criteria as those used in the group differences analysis. The outcome variable was the FM-100 score, while the predictors were the mean DAT-SPECT striatum, caudate, or putamen SBRs from both hemispheres, with age, sex, and education included as covariates.

#### Association of colour discrimination with MRI data

A VBM analysis was performed using a multiple regression model with FM-100 score as the dependent variable and total intracranial volume (TIV), age, sex, and education as covariates of no interest. A full factorial model including the same covariates as in the regression model was used to identify interactions between the patient group and the control group. The statistical map for the correlation analysis was cut at the cluster level at the statistical level of *p* < 0.05.

Group differences in descriptive variables were tested using the Mann–Whitney U test or chi-square test.

In each analysis, except analyses of descriptive variables, *p* values were corrected for multiple comparisons using the Benjamini–Hochberg procedure. However, for transparency, *p* values are reported in the text and tables at their original value and highlighted in bold if they survived multiplicity correction unless otherwise indicated.

Analyses were conducted using R (4.3.3) implemented in RStudio (2024.12.1.563) and CAT12 (12.8.2). All R packages and libraries utilized in this study were maintained up-to-date to ensure compatibility and reliability.

## Results

Seven CONs were excluded from the total cohort due to the presence of MCI, and seven iRBDs were excluded due to the presence of overt parkinsonism. Data were missing for 55 participants (22 CONs and 33 iRBDs). Seven patients had missing DAT-SPECT data, yet were included in other analyses. The final sample consisted of 77 CONs and 73 patients with iRBD. In the iRBD group, 25% of the sample met the criteria for MCI and 23% had abnormal DAT-SPECT. Other descriptive statistics are presented in Table  1. The two groups differed in all clinical and demographic variables. Given this and the assumed causal effect of these variables on the variables of interest, we controlled our main analysis for the effects of age, sex and education.

**Table 1:** Socio-demographic and clinical characteristics of iRBD patients and controls

	CON (*N* = 77)			iRBD (*N* = 73)			*p*
	*M*	*Mdn*	*SD*	*M*	*Mdn*	*SD*	
Age, years	60.08	59.60	10.68	66.60	66.90	7.09	< 0.001
Sex, females %	–	37.66	–	–	8.22	–	< 0.001
Education, years	16.14	17.00	3.50	14.40	13.00	3.19	0.002
RBD duration, years	–	–	–	7.38	5.00	9.00	–
MCI, negative %	–	100	–	–	75.34	–	–
DAT-SPECT, negative %	–	–	–	–	77.27	–	–
SBR putamen	–	–	–	2.44	2.34	0.57	–
SBR caudate	–	–	–	2.84	2.80	0.55	–
MoCA	26.43	26.00	2.00	23.67	24.00	2.83	< 0.001
BDI-II	3.81	3.00	3.63	9.33	8.00	7.80	< 0.001
STAI-X1	30.74	31.00	6.57	36.92	34.00	9.92	< 0.001
STAI-X2	32.13	32.00	6.72	39.39	38.00	9.37	< 0.001
MDS-UPDRS-III	3.86	3.00	3.41	6.16	4.00	5.76	0.016
FM-100 (TES)	50.16	40.00	37.14	83.21	72.00	48.63	< 0.001
FM-100 (RY)	6.68	4.00	8.93	15.34	10.00	19.43	< 0.001
FM-100 (YG)	13.17	12.00	11.33	20.71	20.00	14.03	< 0.001
FM-100 (GB)	18.16	16.00	15.49	27.75	24.00	18.33	< 0.001
FM-100 (BR)	12.16	10.00	10.97	19.40	20.00	11.70	< 0.001

*MCI* mild cognitive impairment; *MoCA* Montreal Cognitive Assessment; *BDI-II* Beck Depression Inventory, Second Edition; *STAI* State-Trait Anxiety Inventory; *MDS-UPDRS-III* Movement Disorders Society-Unified Parkinson’s Disease Rating Scale, part III; *FM-100* Farnsworth-Munsell 100 Hue Colour Vision Test; *TES* total error score; *RY* red-yellow; *BR* blue-red; *YG* yellow-green; *GB* green–blue

### Group differences in colour discrimination

**Fig. 3:**
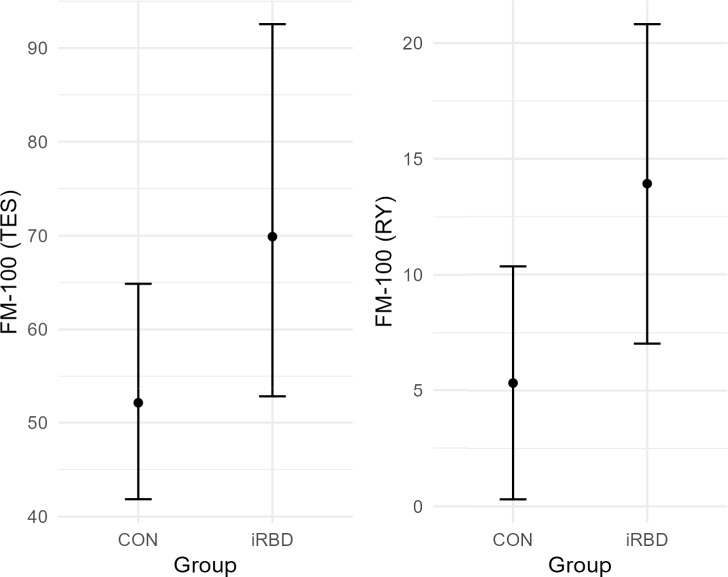
Predicted marginal means of FM-100 by group. *Note*. The whiskers represent the 95% confidence interval. *FM-100* = Farnsworth-Munsell 100 Hue Colour Vision Test; *TES* = total error score; *RY* = red-yellow

iRBD diagnosis was significantly associated with FM-100 (TES) score, *B* = 0.294, *SE* = 0.114, *t* = 2.578, *p* = 0.011, 95% CI[0.070, 0.516], and FM-100 (RY), *B* = 8.599, *SE* = 3.215, *z* = 2.674, *p* = 0.007, keeping demographic variables constant. In other words, iRBDs had approximately 34% worse colour discrimination (TES score) than CONs and scored on average 9 points higher in the red-yellow spectrum, see Table  2 and Fig. 3.

**Table 2:** Group differences in colour discrimination adjusted for age, sex and education

DV	IV	*B*	*SE*	CI		Statistic	*p*
				LL	UL		
FM-100 (TES)	Group	0.294	0.114	0.070	0.516	2.578	**0.011**
FM-100 (BR)	Group	5.139	2.524	0.192	10.087	2.036	0.042
FM-100 (GB)	Group	6.445	3.658	−0.726	13.615	1.762	0.078
FM-100 (RY)	Group	8.599	3.215	2.297	14.900	2.674	**0.007**
FM-100 (YG)	Group	4.359	2.454	−0.451	9.169	1.776	0.076

A generalized linear model with a gamma distribution and a log link function was used for the TES score. For the other scores, a Tobit model with gaussian distribution and robust standard errors was used. For the Tobit statistic = *z*, for the GLM statistic = *t*. *DV* dependent variable; *IV* independent variable; *FM-100* Farnsworth-Munsell 100 Hue Colour Vision Test; *TES* total error score; *RY* red-yellow; *BR* blue-red; *YG* yellow-green; *GB* green–blue

Significant results after correction for multiple comparison are in bold

### Effect of iRBD on colour discrimination through cognition

**Fig. 4:**
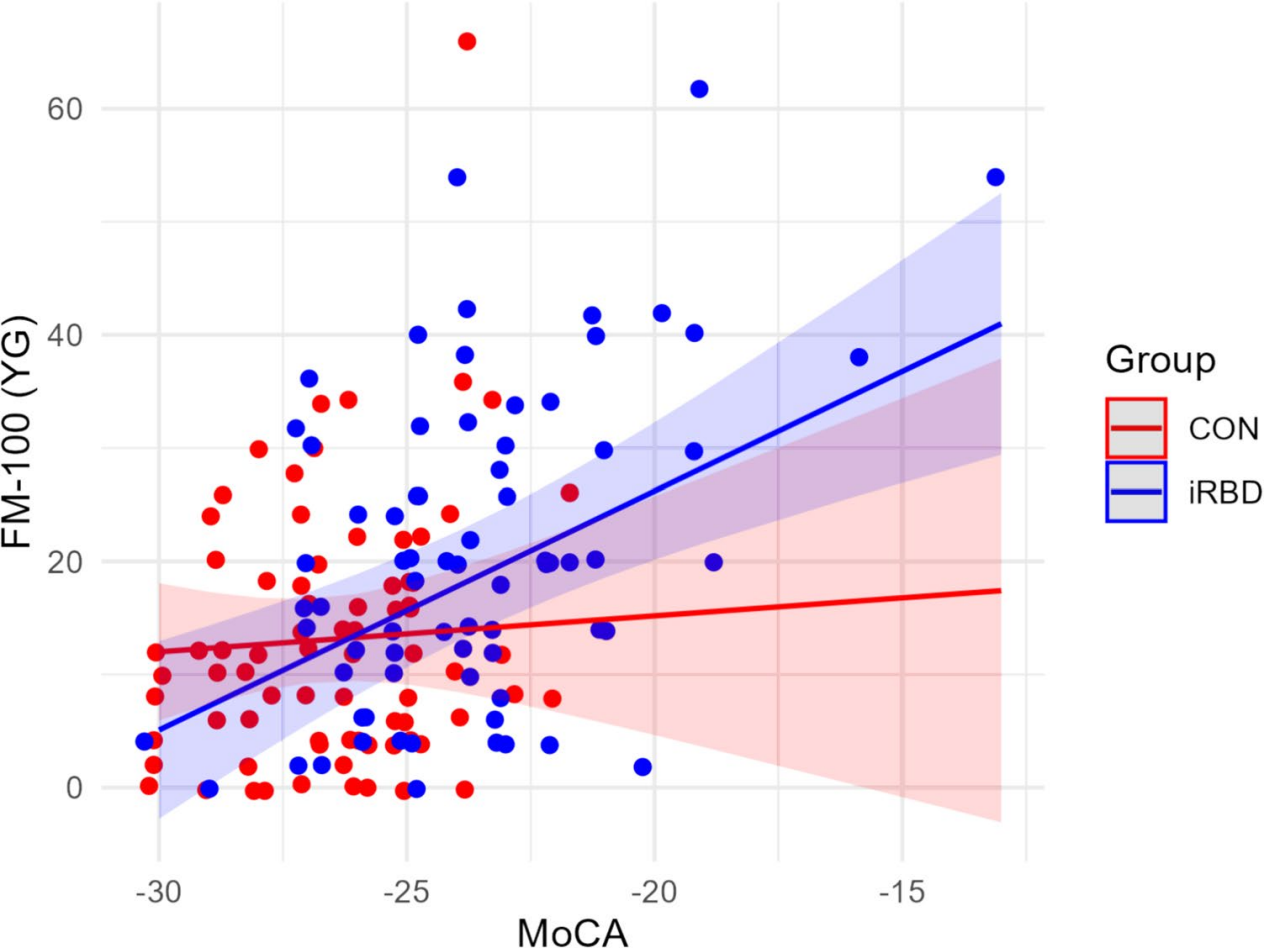
Group × MoCA interaction in mediation analysis of group and FM-100 (YG) with MoCA mediator. *Note.* For better display, the points are depicted with additional variability of .3. *FM-100* = Farnsworth-Munsell 100 Hue Colour Vision Test; *YG* = yellow-green; *MoCA* = Montreal Cognitive Assessment

Analysis of the colour discrimination – cognition pattern showed that there is a mediating effect of group on FM-100 (YG) via MoCA, ACMA = 3.717, *SE* = 1.270, *p* < 0.001, 95% CI [1.480, 6.493], and this effect is significantly different for iRBD from the effects for CON, *t* = 3.189, *p* = 0.041 (Table 3 in appendix, Fig. 4). The mediating effects of the other variables were mostly small, with two exceptions, namely verbal memory (AVLT) and motor symptoms (MDS-UPDRS-III), which were significant before correction for multiple comparisons.

### Association of colour discrimination with dopamine transporter imaging

The putamen, caudate, and striatum were selected as DAT-SPECT ROIs for hypotheses about the role of dopamine in colour discrimination. Of the variables tested, only FM-100 (GB) had a significant association with the caudate SBR, *B* = 15.040, *SE* = 4.795, *z* = 3.137, *p* = 0.002, 95% CI [5.642, 24.438], see Table 4 in appendix.

### Association of colour discrimination with MRI data

**Fig. 5:**
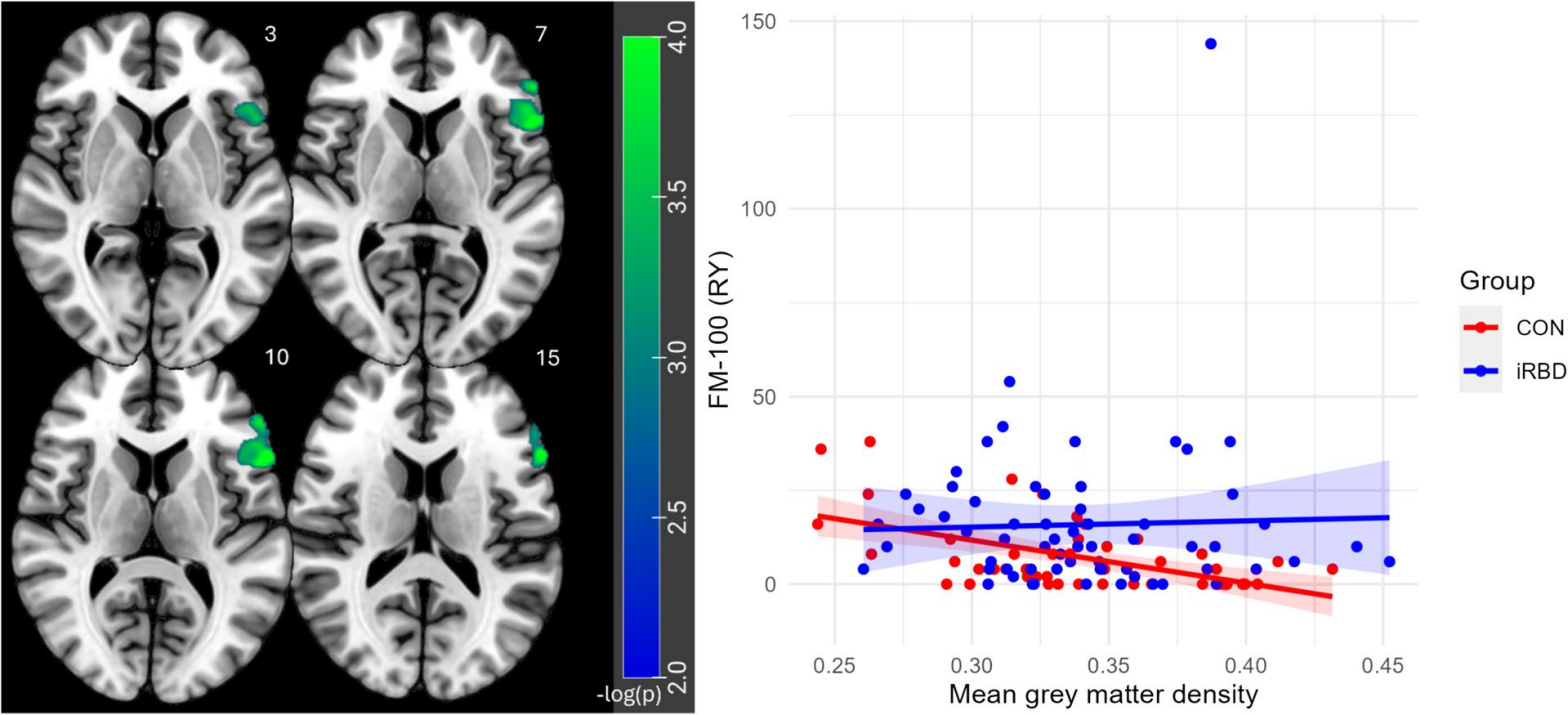
Full factorial analysis of FM-100 scores and group. *Note.* Significant cluster for steeper dependence of iRBD vs. CON with FM-100 (RY). Z-coordinates in the Montreal Neurological Institute space (in millimeters) are indicated next to each slice (top right)

In full factorial analysis, a cluster as shown in Fig. 5 covering regions of left frontal inferior gyrus pars triangularis and opercularis was identified which for FM-100 (RY) showed a positive dependence for CON and none for RBD (*p*_*FWE*_ = 0.014). However, this relationship was largely driven by a single outlier, and upon its removal, the result became nonsignificant (*p*_*FWE*_ = 0.182).

## Discussion

Three main hypotheses have been proposed to explain colour vision deficit in alpha-synucleinopathies, including disruption of the visual system (Unger et al. 2010; Polo et al. 2016), dopaminergic denervation in the brain (Colzato et al. 2014), and diffuse morphological changes (Bertrand et al. 2012). So far, they have been tested independently, in small samples, without a control group, or relying only on total scores. In this study, we tried to overcome these limitations.

Our study is among the few that have specifically analyzed FM-100 subscores, allowing for a more detailed assessment of colour discrimination deficits in iRBD. Along with confirming overall impairment (Postuma et al. 2006, 2009; Dusek et al. 2019; Fereshtehnejad et al. 2019; Li et al. 2019; Kim et al. 2024), we also found differences in specific subscores, enabling a direct comparison with the only two other studies that have examined this level of detail. In our sample, iRBD scored worse than CON in FM-100 (RY), whereas in the Li et al. (2019) study iRBD scored worse in all but the FM-100 (YG), and in the Postuma et al. (2006) study performed worse in all four subscores. All studies indicate a deficit in the red-yellow spectrum, suggesting this spectrum may be the most affected. Considering the number of occurrences across studies, one might assume the most pronounced deficit is in red vision, as red, yellow, green and blue are present in 5, 4, 3 and 4 instances, respectively, among all significant spectra. Nevertheless, there is a lack of robust data and rigorous methods, and some findings suggest the blue spectrum may be more affected (Haug et al. 1995; Sartucci et al. 2003). Additionally, since all studies, except for the present one, report differences across multiple spectra, the deficit seems unlikely to be limited to a single hue.

Mediation analysis was conducted to determine whether cognitive deficits contribute to colour discrimination impairment in iRBD. While one significant indirect effect was found, i.e., iRBD on FM-100 (YG) via MoCA, the overall results did not align with the causal assumptions outlined in Fig. 1c or the a priori criteria mentioned in the Methods section. The primary reason is the absence of an indirect effect on FM-100 scores that would differ between groups, suggesting that cognitive functions did not significantly influence performance on either the FM-100 (TES) or FM-100 (RY). To the best of our knowledge, no previous studies have specifically examined the mediation effects of cognition on colour discrimination in iRBD. However, several studies on alpha-synucleinopathies, including iRBD (Muller et al. 1998; Postuma et al. 2009; Bertrand et al. 2012; Li et al. 2019; Matar et al. 2019; Diederich et al. 2020; Kim et al. 2024), have demonstrated an association between colour discrimination and clinical variables such as motor symptoms, olfaction, or visuo-constructions, often with medium to large effect sizes. Based on our findings, we cannot entirely dismiss the potential role of cognitive functions in the colour discrimination deficits observed in iRBD (as suggested by the significant mediation via MoCA). However, we propose that this influence is likely subtle and does not fully explain the differences between the CON and iRBD.

Based on the predefined criteria for interpretation, we conclude that the FM-100 cannot be considered a reliable proxy for the central dopaminergic state in iRBD. However, the association between FM-100 (GB) and the caudate nucleus binding ratios may suggest some underlying mechanism, as referenced in Colzato et al. (2014). A potential issue with this hypothesis is that the authors of the cited study do not clearly define the concept of the central dopaminergic state. We used the striatum, including the caudate and putamen, as our DAT-SPECT regions of interest, given their innervation by dopaminergic neurons from the substantia nigra and their relevance to Parkinsonian symptoms (Kordower et al. 2013; Purves et al. 2018). Interpreting the association of FM-100 (GB) with the caudate nucleus SBR is more complex. Due to the absence of DAT-SPECT data from the CON group, it is unclear whether this reflects a genuine underlying mechanism or is merely a non-specific association. However, if the colour discrimination deficit were caused by reduced dopamine in the caudate nucleus, which is associated with cognitive functions (Grahn et al. 2008), we would expect to observe a association with more FM-100 scores or at least one significant cognitive mediator for FM-100 (GB) which was not the case in our study. Furthermore, little evidence supports the idea that striatal dopamine depletion directly modulates colour vision. To our knowledge, no human studies have tested this connection, and only one animal study has linked brainstem dopamine to the modulation of visual processing (Govindaiah and Cox 2005).

The VBM analysis revealed a significant interaction for FM-100 (RY) in the left inferior frontal gyrus, specifically in the pars triangularis and opercularis. This finding was unexpected, as we had anticipated involvement in posterior regions typically associated with visual processing. While the left inferior frontal gyrus is commonly linked to language functions (Friederici 2011), there is also evidence supporting its role in colour processing (Roux et al. 2006; Siuda-Krzywicka et al. 2021). In particular, Siuda-Krzywicka et al. (2021) demonstrated that colour categorization is associated with stronger connections between preselected seeds and the inferior frontal gyrus. These authors suggest that colour categorization can be understood within the framework of controlled semantic cognition, which involves the interaction between executive functions and semantics, respectively the interaction of frontal and temporal areas (Siuda-Krzywicka et al. 2021). Although disruption of the above mentioned connections could potentially explain our results, it is more likely that the outcome occurred by chance, as indicated by the analysis that excluded outliers.

The results of the present study are limited in several respects. We lacked a control group for the DAT-SPECT analysis due to ethical and technical constraints. We relied solely on the FM-100 as a measure of colour discrimination, which limits the scope of our findings to this test; nonetheless, we believe the results can be cautiously generalized. Although the neuropsychological battery used was comprehensive, incorporating additional tests could be beneficial, particularly for examining the relationship with perceptual processing. This also applies to the assessment of vision, for example, through optical coherence tomography. Finally, while we accounted for demographic variables in all analyses, there remained baseline differences between the groups in these variables.

In conclusion, this study simultaneously tested multiple hypotheses regarding colour discrimination in iRBD. We found that individuals with iRBD performed worse in colour discrimination compared to CON. No distinct pattern emerged from our analysis, suggesting that the colour discrimination deficit in iRBD is not associated with a single dominant factor, as has sometimes been assumed. Instead, it appears to be influenced by several smaller factors, which may or may not be independent of each other.

## Data Availability

The data that support the findings of this study are available on request from the corresponding author. The data are not publicly available due to privacy or ethical restrictions.

## Appendix

See Tables 3 and 4.

**Table 3:** Indirect effects of group on colour discrimination through cognition based on mediation analysis with cognition × group interaction

Mediator	Outcome	iRBD					CON					CON vs iRBD	
		ACME	*SE*	*p*	CI		ACME	*SE*	*p*	CI		t	*p*
					LL	UL				LL	UL		
AVLT (delayed recall)	FM-100 (TES)	3.603	2.903	0.133	−1.107	10.278	1.830	2.640	0.450	−3.028	7.608		
AVLT (delayed recall)	FM-100 (RY)	1.374	1.060	0.113	−0.331	3.809	0.005	1.036	0.999	−2.063	2.101		
AVLT (delayed recall)	FM-100 (YG)	1.872	1.007	0.012	0.272	4.171	0.956	0.825	0.191	−0.433	2.808		
AVLT (delayed recall)	FM-100 (GB)	0.212	1.096	0.815	−2.026	2.441	0.489	0.870	0.546	−1.131	2.353		
AVLT (delayed recall)	FM-100 (BR)	1.243	0.890	0.089	−0.223	3.252	0.101	0.854	0.896	−1.667	1.866		
AVLT 1–5	FM-100 (TES)	7.585	3.264	0.001	2.188	14.749	1.531	2.152	0.442	−2.371	6.247		
AVLT 1–5	FM-100 (RY)	3.144	2.056	0.058	−0.035	8.064	0.046	0.986	0.959	−1.944	2.105		
AVLT 1–5	FM-100 (YG)	3.216	1.301	0.002	0.915	6.038	0.868	0.802	0.223	−0.494	2.703		
AVLT 1–5	FM-100 (GB)	1.791	1.057	0.023	0.142	4.219	0.234	0.770	0.739	−1.271	1.877		
AVLT 1–5	FM-100 (BR)	2.194	1.032	0.003	0.501	4.580	0.276	0.612	0.632	−0.915	1.603		
CDT	FM-100 (TES)	1.730	1.698	0.230	−0.746	5.724	0.975	2.033	0.599	−2.258	6.154		
CDT	FM-100 (RY)	1.100	1.265	0.305	−0.761	4.250	0.421	0.598	0.442	−0.500	1.906		
CDT	FM-100 (YG)	0.654	0.664	0.251	−0.362	2.214	−0.003	0.561	0.997	−1.208	1.200		
CDT	FM-100 (GB)	0.616	0.823	0.385	−0.668	2.592	0.179	0.699	0.795	−1.140	1.799		
CDT	FM-100 (BR)	0.032	0.450	0.935	−0.918	1.012	−0.061	0.298	0.843	−0.737	0.531		
GPT (left hand)	FM-100 (TES)	−0.107	1.278	0.932	−2.933	2.520	−0.370	3.524	0.911	−7.739	6.444		
GPT (left hand)	FM-100 (RY)	−0.055	0.585	0.921	−1.435	1.175	−0.095	0.775	0.905	−1.697	1.495		
GPT (left hand)	FM-100 (YG)	−0.034	0.325	0.919	−0.802	0.621	−0.081	0.721	0.909	−1.642	1.426		
GPT (left hand)	FM-100 (GB)	−0.024	0.355	0.963	−0.887	0.704	−0.124	1.067	0.906	−2.366	2.061		
GPT (left hand)	FM-100 (BR)	−0.003	0.187	0.983	−0.458	0.376	−0.051	0.508	0.929	−1.169	1.034		
GPT (right hand)	FM-100 (TES)	−1.628	3.621	0.621	−9.806	5.231	−1.238	3.879	0.702	−9.693	4.767		
GPT (right hand)	FM-100 (RY)	−0.625	1.334	0.618	−3.729	1.802	−0.330	0.692	0.615	−1.863	0.946		
GPT (right hand)	FM-100 (YG)	−0.423	0.863	0.607	−2.352	1.166	−0.174	0.463	0.707	−1.271	0.661		
GPT (right hand)	FM-100 (GB)	−0.645	1.384	0.623	−3.928	1.896	−0.476	1.138	0.661	−3.084	1.627		
GPT (right hand)	FM-100 (BR)	−0.223	0.520	0.646	−1.484	0.697	−0.082	0.268	0.773	−0.722	0.406		
LNS	FM-100 (TES)	2.775	2.667	0.193	−0.980	9.176	1.938	1.843	0.181	−0.589	6.465		
LNS	FM-100 (RY)	1.850	1.635	0.167	−0.642	5.862	1.112	0.918	0.133	−0.281	3.258		
LNS	FM-100 (YG)	1.408	1.111	0.131	−0.382	3.983	0.787	0.748	0.203	−0.273	2.637		
LNS	FM-100 (GB)	0.308	0.798	0.651	−1.255	2.087	0.298	0.556	0.566	−0.652	1.627		
LNS	FM-100 (BR)	0.213	0.506	0.629	−0.724	1.403	0.235	0.372	0.485	−0.382	1.151		
MBT (delayed free recall)	FM-100 (TES)	−0.684	1.721	0.655	−4.685	2.402	−0.212	1.166	0.846	−2.951	2.004		
MBT (delayed free recall)	FM-100 (RY)	−0.238	0.629	0.691	−1.745	0.900	−0.176	0.528	0.724	−1.414	0.792		
MBT (delayed free recall)	FM-100 (YG)	−0.153	0.404	0.689	−1.119	0.600	0.065	0.475	0.904	−0.848	1.195		
MBT (delayed free recall)	FM-100 (GB)	−0.223	0.556	0.665	−1.544	0.830	0.019	0.504	0.993	−1.048	1.177		
MBT (delayed free recall)	FM-100 (BR)	−0.202	0.465	0.642	−1.264	0.670	−0.032	0.273	0.899	−0.651	0.545		
MBT (free recall)	FM-100 (TES)	2.828	2.973	0.283	−2.229	9.366	0.172	1.294	0.887	−2.487	3.173		
MBT (free recall)	FM-100 (RY)	0.761	0.948	0.373	−0.701	3.138	0.528	0.680	0.393	−0.481	2.245		
MBT (free recall)	FM-100 (YG)	0.621	0.738	0.349	−0.544	2.366	−0.197	0.542	0.727	−1.516	0.775		
MBT (free recall)	FM-100 (GB)	1.055	1.172	0.315	−0.911	3.799	−0.344	0.655	0.593	−1.937	0.668		
MBT (free recall)	FM-100 (BR)	0.581	0.668	0.329	−0.511	2.189	0.053	0.320	0.863	−0.602	0.787		
MDS-UPDRS-III	FM-100 (TES)	1.706	2.278	0.391	−2.197	6.952	2.028	3.094	0.473	−3.378	8.982		
MDS-UPDRS-III	FM-100 (RY)	0.848	0.700	0.143	−0.188	2.483	1.352	1.370	0.240	−0.850	4.585		
MDS-UPDRS-III	FM-100 (YG)	1.464	0.903	0.039	0.048	3.534	0.436	0.998	0.645	−1.448	2.689		
MDS-UPDRS-III	FM-100 (GB)	−0.012	0.754	0.990	−1.594	1.461	−0.779	1.423	0.565	−3.899	1.824		
MDS-UPDRS-III	FM-100 (BR)	0.045	0.534	0.915	−1.055	1.109	0.175	1.054	0.876	−1.891	2.402		
MoCA	FM-100 (TES)	6.821	3.746	0.045	0.198	14.562	−0.801	3.826	0.797	−8.257	7.180		
MoCA	FM-100 (RY)	5.000	3.607	0.122	−1.546	13.064	0.710	1.609	0.645	−2.493	4.016		
MoCA	FM-100 (YG)	3.717	1.270	** < 0.001**	1.480	6.493	0.528	1.385	0.677	−2.212	3.282	3.189	0.041
MoCA	FM-100 (GB)	1.612	1.248	0.159	−0.676	4.280	−1.280	1.920	0.499	−5.312	2.323		
MoCA	FM-100 (BR)	1.865	1.032	0.046	0.034	4.114	−1.158	1.503	0.435	−4.337	1.635		
MoCA (Cube)	FM-100 (TES)	2.344	2.313	0.249	−1.648	7.523	−0.074	1.630	0.944	−3.519	3.511		
MoCA (Cube)	FM-100 (RY)	1.154	1.436	0.358	−1.086	4.487	0.305	0.513	0.516	−0.509	1.552		
MoCA (Cube)	FM-100 (YG)	0.735	0.819	0.302	−0.636	2.586	0.139	0.788	0.861	−1.468	2.014		
MoCA (Cube)	FM-100 (GB)	0.852	1.106	0.375	−0.857	3.458	−0.124	0.571	0.838	−1.505	1.009		
MoCA (Cube)	FM-100 (BR)	0.714	0.749	0.283	−0.597	2.358	−0.041	0.274	0.896	−0.682	0.514		
PST (colors)	FM-100 (TES)	2.579	2.828	0.276	−1.869	9.400	0.774	2.074	0.707	−2.790	5.888		
PST (colors)	FM-100 (RY)	0.320	0.560	0.547	−0.603	1.743	0.157	0.716	0.805	−1.303	1.864		
PST (colors)	FM-100 (YG)	0.444	0.558	0.367	−0.393	1.846	0.508	0.699	0.428	−0.557	2.350		
PST (colors)	FM-100 (GB)	1.087	1.027	0.229	−0.632	3.382	0.154	0.867	0.841	−1.692	2.260		
PST (colors)	FM-100 (BR)	0.986	0.965	0.234	−0.603	3.262	−0.062	0.510	0.911	−1.237	1.020		
PST (dots)	FM-100 (TES)	2.715	2.368	0.154	−0.834	8.116	3.622	3.353	0.165	−1.143	11.659		
PST (dots)	FM-100 (RY)	0.988	0.875	0.181	−0.374	3.045	1.127	1.011	0.196	−0.402	3.590		
PST (dots)	FM-100 (YG)	0.757	0.697	0.199	−0.295	2.394	0.918	0.954	0.260	−0.480	3.276		
PST (dots)	FM-100 (GB)	0.857	0.855	0.237	−0.380	2.911	0.766	1.038	0.414	−0.855	3.300		
PST (dots)	FM-100 (BR)	1.002	0.848	0.171	−0.377	2.906	0.782	0.752	0.221	−0.322	2.587		
PST (interference)	FM-100 (TES)	2.903	3.061	0.260	−2.100	9.951	1.777	2.249	0.323	−1.436	7.192		
PST (interference)	FM-100 (RY)	1.058	1.177	0.297	−0.870	3.822	0.997	1.073	0.292	−0.720	3.563		
PST (interference)	FM-100 (YG)	1.106	1.058	0.271	−0.849	3.289	−0.044	0.420	0.939	−1.020	0.825		
PST (interference)	FM-100 (GB)	0.632	0.835	0.399	−0.686	2.684	0.572	0.953	0.511	−0.965	3.041		
PST (interference)	FM-100 (BR)	0.556	0.631	0.304	−0.467	2.042	0.611	0.639	0.285	−0.443	2.100		
PST (words)	FM-100 (TES)	−2.718	5.151	0.575	−14.377	6.162	−0.619	1.462	0.644	−4.137	1.737		
PST (words)	FM-100 (RY)	−0.534	0.925	0.532	−2.743	1.089	−0.341	0.534	0.500	−1.511	0.653		
PST (words)	FM-100 (YG)	−0.756	1.188	0.498	−3.368	1.425	−0.210	0.394	0.561	−1.138	0.465		
PST (words)	FM-100 (GB)	−0.784	1.308	0.514	−3.848	1.543	−0.001	0.254	0.983	−0.580	0.562		
PST (words)	FM-100 (BR)	−0.751	1.212	0.501	−3.527	1.401	−0.275	0.422	0.499	−1.186	0.517		
SDMT	FM-100 (TES)	3.660	3.045	0.180	−1.523	10.315	1.012	2.378	0.639	−3.342	6.717		
SDMT	FM-100 (RY)	1.462	1.135	0.147	−0.465	4.063	0.583	0.816	0.419	−0.693	2.601		
SDMT	FM-100 (YG)	1.252	0.961	0.147	−0.410	3.391	0.423	0.615	0.440	−0.557	1.942		
SDMT	FM-100 (GB)	1.578	1.251	0.147	−0.501	4.415	−0.021	0.686	0.981	−1.495	1.474		
SDMT	FM-100 (BR)	0.892	0.731	0.150	−0.278	2.573	0.008	0.771	0.999	−1.633	1.732		
TMT-A	FM-100 (TES)	1.768	3.340	0.576	−4.189	9.337	2.108	5.852	0.621	−6.324	14.225		
TMT-A	FM-100 (RY)	0.449	0.882	0.583	−1.155	2.478	0.526	1.102	0.621	−1.406	3.234		
TMT-A	FM-100 (YG)	0.474	0.854	0.567	−1.184	2.280	0.347	0.745	0.638	−0.942	2.202		
TMT-A	FM-100 (GB)	0.600	1.125	0.571	−1.502	3.066	0.437	1.048	0.677	−1.387	3.088		
TMT-A	FM-100 (BR)	0.280	0.544	0.585	−0.734	1.488	0.240	0.590	0.679	−0.793	1.720		
TMT-B	FM-100 (TES)	3.332	2.637	0.107	−0.600	9.782	5.146	9.642	0.323	−2.744	25.089		
TMT-B	FM-100 (RY)	1.878	1.706	0.156	−0.469	6.068	2.179	1.509	0.103	−0.389	5.491		
TMT-B	FM-100 (YG)	1.364	0.957	0.103	−0.290	3.472	0.704	0.926	0.367	−0.759	3.003		
TMT-B	FM-100 (GB)	0.816	0.796	0.200	−0.331	2.750	0.462	0.993	0.589	−1.421	2.860		
TMT-B	FM-100 (BR)	0.230	0.313	0.403	−0.283	0.997	0.824	0.686	0.133	−0.181	2.459		
VF (animals/clothes)	FM-100 (TES)	1.563	2.126	0.417	−1.801	6.651	1.924	2.326	0.367	−2.041	7.232		
VF (animals/clothes)	FM-100 (RY)	0.785	1.305	0.509	−1.264	4.148	0.698	0.828	0.343	−0.695	2.623		
VF (animals/clothes)	FM-100 (YG)	0.647	0.816	0.371	−0.689	2.621	0.137	0.318	0.663	−0.419	0.930		
VF (animals/clothes)	FM-100 (GB)	0.499	0.704	0.433	−0.616	2.241	0.365	0.629	0.540	−0.598	1.944		
VF (animals/clothes)	FM-100 (BR)	0.273	0.461	0.527	−0.457	1.431	0.548	0.640	0.342	−0.550	2.016		
VF (vegetables)	FM-100 (TES)	1.123	2.005	0.531	−2.277	5.877	0.410	0.935	0.649	−1.120	2.727		
VF (vegetables)	FM-100 (RY)	0.051	0.440	0.935	−0.877	1.128	0.137	0.412	0.751	−0.555	1.165		
VF (vegetables)	FM-100 (YG)	0.404	0.758	0.570	−0.943	2.171	0.113	0.332	0.737	−0.463	0.948		
VF (vegetables)	FM-100 (GB)	0.531	1.003	0.573	−1.288	2.866	0.126	0.354	0.721	−0.466	0.998		
VF (vegetables)	FM-100 (BR)	0.222	0.469	0.631	−0.588	1.373	0.101	0.284	0.723	−0.371	0.823		
VF (verbs)	FM-100 (TES)	0.645	1.753	0.701	−2.652	4.536	0.355	1.187	0.775	−1.684	3.191		
VF (verbs)	FM-100 (RY)	0.261	0.985	0.813	−1.507	2.791	0.066	0.447	0.896	−0.766	1.141		
VF (verbs)	FM-100 (YG)	0.260	0.703	0.695	−1.083	1.857	0.026	0.300	0.949	−0.584	0.728		
VF (verbs)	FM-100 (GB)	0.078	0.375	0.866	−0.615	1.000	−0.019	0.371	0.979	−0.838	0.774		
VF (verbs)	FM-100 (BR)	0.128	0.393	0.750	−0.589	1.069	0.091	0.310	0.765	−0.484	0.864		

CON vs iRBD represents testing for differences between treatment and control conditions in average causal mediation effects and direct effects. *ACME* average causal mediation effects; *FM-100* Farnsworth-Munsell 100 Hue Colour Vision Test; *TES* total error score; *RY* red-yellow; *BR* blue-red; *YG* yellow-green; GB green–blue; *MoCA* Montreal Cognitive Assessment; *RAVLT* Rey Auditory Verbal Learning Test; *MBT* Memory Binding Test; *MIST* abbreviated Memory for Intentions Screening Test; *TMT* = Trail Making Test; *LNS* Letter-Number Sequencing from Wechsler Adult Intelligence Scale, Third Revision; *PST* Prague Stroop Test; *VF* Verbal fluency; *CDT* Clock Drawing Test; *GPT* Grooved Pegboard Test; *SDMT* Symbol Digit Modalities Test. Significant results after correction are in bold

**Table 4:** Relationship of colour discrimination and DAT-SPECT values adjusted for education, sex and age

DV	IV	*B*	*SE*	CI		Statistic	*p*
				LL	UL		
FM-100 (BR)	Nucleus caudatus	3.423	2.344	−1.170	8.017	1.461	0.144
FM-100 (BR)	Striatum	3.423	2.516	−1.508	8.354	1.361	0.174
FM-100 (BR)	Putamen	3.107	2.571	−1.932	8.146	1.208	0.227
FM-100 (GB)	Nucleus caudatus	15.040	4.795	5.642	24.438	3.137	0**.002**
FM-100 (GB)	Striatum	13.232	5.231	2.980	23.485	2.530	0.011
FM-100 (GB)	Putamen	11.397	5.417	0.780	22.014	2.104	0.035
FM-100 (RY)	Nucleus caudatus	7.035	3.944	−0.694	14.764	1.784	0.074
FM-100 (RY)	Striatum	4.879	4.278	−3.505	13.263	1.141	0.254
FM-100 (RY)	Putamen	3.688	4.292	−4.724	12.099	0.859	0.390
FM-100 (TES)	Nucleus caudatus	0.277	0.114	0.069	0.490	2.438	0.018
FM-100 (TES)	Striatum	0.229	0.119	0.010	0.457	1.925	0.059
FM-100 (TES)	Putamen	0.191	0.118	−0.026	0.416	1.619	0.111
FM-100 (YG)	Nucleus caudatus	3.576	3.390	−3.067	10.220	1.055	0.291
FM-100 (YG)	Striatum	2.144	3.404	−4.528	8.817	0.630	0.529
FM-100 (YG)	Putamen	1.409	3.297	−5.052	7.870	0.427	0.669

A generalized linear model with a gamma distribution and a log link function was used for the TES score. For the other scores, a Tobit model with gaussian distribution and robust standard errors was used. For the Tobit statistic = *z*, for the GLM statistic = *t*. *DV* dependent variable; *IV* independent variable; *FM-100* Farnsworth-Munsell 100 Hue Colour Vision Test; *TES* total error score; *RY* red-yellow; *BR* blue-red; *YG* yellow-green; *GB* green–blue

Significant results after correction are in bold

## References

[CR1] American Academy of Sleep Medicine (2014) International classification of sleep disorders, 3rd edn. American Academy of Sleep Medicine, Darien IL, p 456

[CR2] American Educational Research Association, American Psychological Association, National Council on Measurement in Education (2014) Standards for educational and psychological testing. American Educational Research Association, Washington DC, p 567

[CR3] Arnaldi D, Mattioli P, Raffa S et al (2024) Presynaptic dopaminergic imaging characterizes patients with REM sleep behavior disorder due to synucleinopathy. Ann Neurol 95:1178–1192. 10.1002/ana.26902 38466158 10.1002/ana.26902 PMC11102309

[CR4] Beck AT, Steer RA, Brown GK (1996) Manual for the beck depression inventory-II. Psychological Corporation, San Antonio, TX

[CR5] Bertrand JA, Bedetti C, Postuma RB et al (2012) Color discrimination deficits in parkinson’s disease are related to cognitive impairment and white-matter alterations. Mov Disord 27:1781–1788. 10.1002/mds.25272 23147270 10.1002/mds.25272

[CR6] Bezdicek O, Motak L, Axelrod BN et al (2012) Czech version of the trail making test: normative data and clinical utility. Arch Clin Neuropsych 27:906–914. 10.1093/arclin/acs084 10.1093/arclin/acs084 23027441

[CR7] Bezdicek O, Raskin SA, Altgassen M, Ruzicka E (2014a) Assessment of prospective memory—a validity study of memory for intentions screening test. Cesk Slov Neurol N 77:435–443

[CR8] Bezdicek O, Stepankova H, Moták L et al (2014b) Czech version of rey auditory verbal learning test: normative data. Aging Neuropsychol Cogn 21:693–721. 10.1080/13825585.2013.865699 10.1080/13825585.2013.865699 24344673

[CR9] Bezdicek O, Lukavsky J, Stepankova H et al (2015) The prague stroop test: Normative standards in older czech adults and discriminative validity for mild cognitive impairment in parkinson’s disease. J Clin Exp Neuropsyc 37:794–807. 10.1080/13803395.2015.1057106 10.1080/13803395.2015.1057106 26313510

[CR10] Buschke H (2014) Rationale of the memory binding test. Dementia and memory. Psychology Press, New York, NY, US, pp 55–71

[CR11] Ciharova M, Cigler H, Dostalova V et al (2020) Beck depression inventory, czech version: demographic correlates, factor structure and comparison with foreign data. Int J Psychiatry Clin Pract 24:371–379. 10.1080/13651501.2020.1775854 32552177 10.1080/13651501.2020.1775854

[CR12] Colzato LS, Sellaro R, Hulka LM et al (2014) Cognitive control predicted by color vision, and vice versa. Neuropsychologia 62:55–59. 10.1016/j.neuropsychologia.2014.07.010 25058057 10.1016/j.neuropsychologia.2014.07.010

[CR13] Dall’Antonia I, Šonka K, Dušek P (2018) Olfaction and colour vision: What can they tell us about parkinson’s disease? Prague Med Rep 119:85–96. 10.14712/23362936.2018.8 10.14712/23362936.2018.8 30414359

[CR14] Darcourt J, Booij J, Tatsch K et al (2010) EANM procedure guidelines for brain neurotransmission SPECT using (123)i-labelled dopamine transporter ligands, version 2. Eur J Nucl Med Mol Imaging 37:443–450. 10.1007/s00259-009-1267-x 19838702 10.1007/s00259-009-1267-x

[CR15] Diederich NJ, Sauvageot N, Pieri V et al (2020) The clinical non-motor connectome in early parkinson’s disease. J Parkinsons Dis 10:1797–1806. 10.3233/JPD-202102 32925095 10.3233/JPD-202102 PMC7683075

[CR16] Dusek P, Ibarburu V, Bezdicek O et al (2019) Relations of non-motor symptoms and dopamine transporter binding in REM sleep behavior disorder. Sci Rep 9:15463. 10.1038/s41598-019-51710-y 31664065 10.1038/s41598-019-51710-y PMC6820530

[CR17] Farnsworth D (1957) The farnsworth-munsell 100-hue test: manual. Munsell Color Company, New York

[CR18] Fereshtehnejad S-M, Yao C, Pelletier A et al (2019) Evolution of prodromal parkinson’s disease and dementia with lewy bodies: A prospective study. Brain 142:2051–2067. 10.1093/brain/awz111 31111143 10.1093/brain/awz111

[CR19] Friederici AD (2011) The brain basis of language processing: From structure to function. Physiol Rev 91:1357–1392. 10.1152/physrev.00006.2011 22013214 10.1152/physrev.00006.2011

[CR20] Goetz CG, Tilley BC, Shaftman SR et al (2008) Movement disorder society-sponsored revision of the unified parkinson’s disease rating scale (MDS-UPDRS): Scale presentation and clinimetric testing results. Mov Disord 23:2129–2170. 10.1002/mds.22340 19025984 10.1002/mds.22340

[CR21] Govindaiah G, Cox CL (2005) Excitatory actions of dopamine via D1-like receptors in the rat lateral geniculate nucleus. J Neurophysiol 94:3708–3718. 10.1152/jn.00583.2005 16107529 10.1152/jn.00583.2005

[CR22] Grahn JA, Parkinson JA, Owen AM (2008) The cognitive functions of the caudate nucleus. Prog Neurobiol 86:141–155. 10.1016/j.pneurobio.2008.09.004 18824075 10.1016/j.pneurobio.2008.09.004

[CR23] Haug BA, Kolle RU, Trenkwalder C et al (1995) Predominant affection of the blue cone pathway in parkinson’s disease. Brain 118(Pt 3):771–778. 10.1093/brain/118.3.771 7600093 10.1093/brain/118.3.771

[CR24] Kim S, Choi JH, Woo KA et al (2024) Clinical correlates of pareidolias and color discrimination deficits in idiopathic REM sleep behavior disorder and parkinson’s disease. J Neural Transm 131:141–148. 10.1007/s00702-023-02724-4 38110521 10.1007/s00702-023-02724-4

[CR25] Kløve H (1963) Clinical neuropsychology. In: Forster FM (ed) The medical clinics of north america. Saunders, New York, NY, pp 1647–1658 14078168

[CR26] Kopecek M, Stepankova H, Lukavsky J et al (2016) Montreal cognitive assessment (MoCA): Normative data for old and very old czech adults. Appl Neuropsychol Adult 24:23–29. 10.1080/23279095.2015.1065261 27144665 10.1080/23279095.2015.1065261

[CR27] Kordower JH, Olanow CW, Dodiya HB et al (2013) Disease duration and the integrity of the nigrostriatal system in parkinson’s disease. Brain 136:2419–2431. 10.1093/brain/awt192 23884810 10.1093/brain/awt192 PMC3722357

[CR28] Li Y, Zhang H, Mao W et al (2019) Visual dysfunction in patients with idiopathic rapid eye movement sleep behavior disorder. Neurosci Lett 709:134360. 10.1016/j.neulet.2019.134360 31269466 10.1016/j.neulet.2019.134360

[CR29] Litvan I, Goldman JG, Tröster AI et al (2012) Diagnostic criteria for mild cognitive impairment in parkinson’s disease: movement disorder society task force guidelines. Mov Disord 27:349–356. 10.1002/mds.24893 22275317 10.1002/mds.24893 PMC3641655

[CR30] Marques A, Dujardin K, Boucart M et al (2010) REM sleep behaviour disorder and visuoperceptive dysfunction: a disorder of the ventral visual stream? J Neurol 257:383–391. 10.1007/s00415-009-5328-7 19789940 10.1007/s00415-009-5328-7

[CR31] Matar E, Phillips JR, Martens KAE et al (2019) Impaired color discrimination—a specific marker of hallucinations in lewy body disorders. J Geriatr Psych Neur 32:257–264. 10.1177/0891988719845501 10.1177/0891988719845501 31035850

[CR32] Muller T, Kuhn W, Buttner T et al (1998) Colour vision abnormalities do not correlate with dopaminergic nigrostriatal degeneration in parkinson’s disease. J Neurol 245:659–664. 10.1007/s004150050263 9776465 10.1007/s004150050263

[CR33] Nasreddine ZS, Phillips NA, Bedirian V et al (2005) The montreal cognitive assessment, MoCA: a brief screening tool for mild cognitive impairment. J Am Geriatr Soc 53:695–699. 10.1111/j.1532-5415.2005.53221.x 15817019 10.1111/j.1532-5415.2005.53221.x

[CR34] Nikolai T, Štěpánková H, Michalec J et al (2015) Testy verbální fluence, česká normativní studie pro osoby vyššího věku. Cesk Slov Neurol N 78(111):292–299. 10.14735/amcsnn2015292

[CR35] Ortuno-Lizaran I, Sanchez-Saez X, Lax P et al (2020) Dopaminergic retinal cell loss and visual dysfunction in parkinson disease. Ann Neurol 88:893–906. 10.1002/ana.25897 32881029 10.1002/ana.25897 PMC10005860

[CR36] Polo V, Satue M, Rodrigo MJ et al (2016) Visual dysfunction and its correlation with retinal changes in patients with parkinson’s disease: an observational cross-sectional study. BMJ Open 6:e009658. 10.1136/bmjopen-2015-009658 10.1136/bmjopen-2015-009658 PMC4861131 27154474

[CR37] Postuma RB, Lang AE, Massicotte-Marquez J, Montplaisir J (2006) Potential early markers of parkinson disease in idiopathic REM sleep behavior disorder. Neurology 66:845–851. 10.1212/01.wnl.0000203648.80727.5b 16567700 10.1212/01.wnl.0000203648.80727.5b

[CR38] Postuma RB, Gagnon JF, Vendette M, Montplaisir JY (2009) Markers of neurodegeneration in idiopathic rapid eye movement sleep behaviour disorder and parkinson’s disease. Brain 132:3298–3307. 10.1093/brain/awp244 19843648 10.1093/brain/awp244

[CR39] Postuma RB, Gagnon JF, Bertrand JA et al (2015) Parkinson risk in idiopathic REM sleep behavior disorder: preparing for neuroprotective trials. Neurology 84:1104–1113. 10.1212/WNL.0000000000001364 25681454 10.1212/WNL.0000000000001364 PMC4371408

[CR40] Purves D, Augustine GJ, Fitzpatrick D et al (2018) Neuroscience, 6th edn. Oxford University Press, Sunderland, MA

[CR41] Rahayel S, Postuma RB, Montplaisir J et al (2018) Cortical and subcortical gray matter bases of cognitive deficits in REM sleep behavior disorder. Neurology 90:e1759–e1770. 10.1212/WNL.0000000000005523 29669906 10.1212/WNL.0000000000005523 PMC5957304

[CR42] Raskin S, Buckheit C, Sherrod C (2010) Memory for intentions test Professional manual. Psychological Assessment Resources, Lutz

[CR43] Roux FE, Lubrano V, Lauwers-Cances V et al (2006) Category-specific cortical mapping: Color-naming areas. J Neurosurg 104:27–37. 10.3171/jns.2006.104.1.27 16509144 10.3171/jns.2006.104.1.27

[CR44] Sartucci F, Orlandi G, Lucetti C et al (2003) Changes in pattern electroretinograms to equiluminant red-green and blue-yellow gratings in patients with early parkinson’s disease. J Clin Neurophysiol 20:375–381. 10.1097/00004691-200309000-00010 14701999 10.1097/00004691-200309000-00010

[CR45] Siuda-Krzywicka K, Witzel C, Bartolomeo P, Cohen L (2021) Color naming and categorization depend on distinct functional brain networks. Cereb Cortex 31:1106–1115. 10.1093/cercor/bhaa278 32995838 10.1093/cercor/bhaa278

[CR46] Smith A (1982) Symbol digits modalities test: Manual. Western Psychological Services, Los Angeles, CA

[CR47] Spielberger CD, Gorsuch RL, Lushene RD et al (1983) Manual for the state-trait anxiety inventory. Consulting Psychologists Press, Palo Alto, CA

[CR48] Therneau TM (2024) A package for survival analysis in R. Version 3.6-4. [computer program]. https://cran.r-project.org/package=survival

[CR49] Tingley D, Yamamoto T, Hirose K et al (2014) mediation: R package for causal mediation analysis. J Stat Softw 59:1–38. 10.18637/jss.v059.i0526917999

[CR50] Unger MM, Belke M, Menzler K et al (2010) Diffusion tensor imaging in idiopathic REM sleep behavior disorder reveals microstructural changes in the brainstem, substantia nigra, olfactory region, and other brain regions. Sleep 33:767–773. 10.1093/sleep/33.6.767 20550017 10.1093/sleep/33.6.767 PMC2881532

[CR51] Wechsler D (2010) Wechslerova inteligenční škála pro dospělé. Hogrefe - Testcentrum, Praha

[CR52] Youn S, Kim T, Yoon IY et al (2016) Progression of cognitive impairments in idiopathic REM sleep behaviour disorder. J Neurol Neurosurg Psychiatry 87:890–896. 10.1136/jnnp-2015-311437 26361986 10.1136/jnnp-2015-311437

[CR53] Zarkali A, McColgan P, Leyland L et al (2021) Visual dysfunction predicts cognitive impairment and white matter degeneration in parkinson’s disease. Movement Disord 36:1191–1202. 10.1002/mds.28477 33421201 10.1002/mds.28477 PMC8248368

